# Mediterranean Diet, Physical Fitness and Body Composition in Sevillian Adolescents: A Healthy Lifestyle

**DOI:** 10.3390/nu11092009

**Published:** 2019-08-26

**Authors:** Pablo Galan-Lopez, Antonio J. Sánchez-Oliver, Francis Ries, José Antonio González-Jurado

**Affiliations:** 1 Physical Education and Sports, Faculty of Educational Sciences, University of Seville (Research Lab HUM 962: Sports and Society), 41013 Seville, Spain; 2 Human Motricity and Sports Performance Area, University of Seville, 41013 Seville, Spain; 3 Faculty of Sports Sciencies, Pablo de Olavide University, 41013 Seville, Spain

**Keywords:** lifestyle, adolescents, sedentary, physical inactivity, nutrition, obesity, health

## Abstract

Childhood and adolescent obesity has become one of the most vital challenges to overcome in the present age. Physical fitness, physical activity and the Mediterranean diet (MD) are valuable tools for its prevention and treatment. The main objective of this study is to analyze the associations between health-related physical fitness components, body composition and adherence to the MD in 917 adolescents aged from 13- to 16-years-old. The ALPHA-Fitness Test was used to measure physical fitness and body composition, and the Adherence to the Mediterranean Diet (KIDMED) questionnaire was employed to assess the adherence to the MD. The associations between variables were tested according to gender and age a generalized linear model (GLM) univariate analysis (two factors) and one-way analysis of variance (ANOVA, with Bonferroni posthoc). As to the body composition and physical fitness variables, significant differences were obtained in both genders but not in relation to the adherence to the MD. The boys performed better in the physical fitness tests. Age was a determinant factor in adherence to the MD in the total sample, lowering as the age of the sample increases. Both the boys and girls who had a significantly higher performance in the endurance test were those who showed high/medium adherence to the MD. It is concluded that higher levels of cardiovascular endurance in boys and girls are associated with a medium and high adherence to the MD.

## 1. Introduction

Lifestyle is defined as all the characteristics of the behavior of a particular person or community [1]. Lifestyle behaviors include a series of habits relevant for health: physical activity, diet, tobacco, alcohol, sleep, etc. [2].

Physical activity and adequate nutrition are among the most determinant habits of a healthy lifestyle [3]. Physical activity, practiced regularly, has significant benefits for health in children and adolescents [4,5,6]. In relation to this, physical fitness, mainly cardiorespiratory fitness, muscular aptitude and motor competence, have been demonstrated to be potent health indicators in children and adolescents [7]. Moreover, low levels of physical activity in young people are directly associated with a series of metabolic risk factors that may persist until adulthood. These are determinant factors in preventive health [8,9].

The Mediterranean diet (MD), characterized by high consumption of whole grains, legumes, fresh fruits, vegetables and olive oil, moderate to high consumption of fish, moderate consumption of dairy products, and low consumption of red meat products, has been accepted as one of the healthiest dietary patterns in the world [10]. Establishing healthy nutritional behaviors is also important during adolescence, as healthy nutrition practices started in this period normally persist in adulthood [11]. Among the different dietary patterns that can be found, the MD is accepted as one of the healthiest dietary models [12], showing a high effectiveness and protection against mortality and morbidity in subjects with a high level of adherence [13,14,15,16]. The studies centered on how the influence of this dietary pattern in children and adolescents has increased in the last few years. In general, it has been shown that adherence to the MD is associated with a reduction of different factors associated with obesity, in addition to high levels of quality of life and better physical and mental health [10,17]. Also, a recent systematic review of the MD adherence in children and adolescents concluded that most of the existing studies showed a direct association between adherence to the MD and physical and inverse activity with sedentary behavior; while results for gender, age, socioeconomic status, and weight were inconsistent [18].

There is increasing evidence that health-related behaviors are grouped [19]. For instance, combining healthy eating habits and regular physical activity is effective in maintaining and improving health, as well as better mental and physical well-being [4]. In the years of growing and development, a combination of regular physical exercise and a healthy diet should be opted for to increase the probability of acquiring a healthy pattern of constant physical maturing [20,21]. Childhood and adolescence are key stages in acquiring the previously mentioned habits, affecting adulthood [22]. Despite the aforementioned benefits, contemporary literature presents patterns of physical activity and diet that are far from healthy standards, being worse in the transition from childhood to adolescence [23]. These data contribute substantially to global mortality, disability and morbidity and increase the prevalence of overweight and obesity in children and adolescents [24,25]. This has been attributed to a great extent to a nutritional transition and the changes of lifestyle characterized by alterations in the supply and intake of foods and the reduction of free time and workplace physical activity [26]. Thus, in this way, physical activity and diet are seen as key elements in the prevention and control of obesity [27,28].

Health-related risks and their evaluation and supervision in these age stages are fundamental for the assigning of resources and the planning of health services and preventative interventions [29], enabling the identification of groups of population and regional areas where the need may be greater [30]. Furthermore, this age stage is fundamental due to the establishment of life habits and to the great number of physical and psychological changes in which they are developed [31,32]. The aim of this study is to examine the physical fitness related with health and adherence to the MD of adolescents in the city of Seville (Spain).

## 2. Materials and Methods

### 2.1. Study and Sample Design

This is a is cross-sectional study that included 13- to 16-years-old students from public and associated centers in Seville, Spain. For the selection of the sample, a confidence interval of 95% with a 10% margin of error was estimated, which was carried out by convenience sampling. A total of 991 participants (491 boys and 500 girls) were selected for this research. Finally, 917 teenagers (458 boys (49.9%), M_age_ = 14.82, standard deviation (SD) = 1.11 and 459 girls (50.1%), M_age_ = 14.83, SD = 1.09) took part in it, which meant a participation rate of 92.53%.

The participants were selected from five schools in the city of Seville: Safa Blanca Paloma (6.8%), La Salle Felipe Benito (18.2%), Salesianos de la Trinidad (20%), Salesianas de Nervión (16.5%) and IES Isbilya (38.6%). The inclusion criteria established for the present research were: participants (boys and girls) aged 13- to 16-years-old; having given informed consent signed by their parents/tutors. Related to their health status, the participants in this research took part regularly in physical education classes without any type of inconvenience. They did not have any kind of cognitive or physical/motor limitation. The Website of Biomedical Research Ethics of Andalusia approved this study (Ref.: 0310-N-17). This guaranteed that all the actions related with this investigation that involved human beings were performed securely. The participants were asked for an oral confirmation to participate and were also informed about their voluntary participation and the possibility of dropping out of the research at any moment.

### 2.2. Instruments

#### 2.2.1. Questionnaire of Adherence to the Mediterranean Diet (KIDMED) 

Adherence to the Mediterranean Diet Questionnaire (KIDMED): the KIDMED questionnaire was used to assess the adherence to the MD in adolescents (http://www. aulamedica.es/nh/pdf/9828.pdf). The questionnaire (previously validated), made up by 16 questions, 12 of them suppose a positive score in relation to the adherence to the MD and, and the other 4 suppose a negative score. Positive answers to the questions that involve greater adherence to the diet are worth +1 point. Positive answers to the questions that suppose fewer adherences to the diet are worth −1 point. Negative answers do not score (a value of 0 was recorded) [33]. KIDMED index is the result of the sum of the scores and goes from 0 to 12 (minimum to maximum adherence). Adherence to MD can be classified into three categories: Low adherence: very low-quality diet (0 to 3 points); Medium adherence: improvement of the dietary pattern is needed (4 to 7 points); and High adherence: optimal MD (8 to 12 points) [34].

#### 2.2.2. Alpha Fitness Battery Test

Alpha-Fitness Battery Test: physical fitness and anthropometric variables were assessed by means of a modified version of the extended ALPHA-Fitness Battery (Ref: 2006120). For measures of physical fitness and body composition parameters, the protocol marked on the ALPHA-Fitness Battery was followed at all times [35] and, as part of a larger research project, all the instruments employed and procedures performed used can be found at Galan-Lopez et al. (2018) [36]. Skin folds were neglected due to time restrictions, body fat percentage (BF%) was measured by bioelectrical impedance (Tanita Inner Scan BF-689, Tanita, Tokyo, Japan) validated by the Food and Drugs administration (FDA, EEUU). Additionally, the 4 × 10 m speed-agility test was employed in order to obtain more data about physical fitness; cardiovascular endurance was evaluated via the 20 m shuttle test [37,38]. The maximum force of the higher extremities was obtained via the force of manual grip, using a hand dynamometer with an adjustable fit (TKK 5401 Grip D, Takey, Tokyo, Japan). 

### 2.3. Methodology

The ALPHA-Fitness Battery Test and the KIDMED questionnaire were completed by all the subjects during their physical education classes. The test battery was organised as a circuit and the different tests were performed successively. The cardiovascular endurance test was done by several students at the same time on a different class day. The preparation and the implementation of all the physical tests took 90 min for each group of students. 

### 2.4. Data Analysis

The data of the descriptive analysis have been presented as a mean (M) + standard deviation (SD). The Kolmogorov-Smirnoff test and Levene test was used to check the normality and homoscedasticity of the variables. To compare the differences between boys and girls a generalized linear model (GLM) multivariate with age as covariate (ANCOVA), and Student’s *t*-test for independent samples was used to compare the age inter-gender. In addition, GLM univariate analysis (two factors) was performed to compare the MD adherence differences by gender and age. In addition, to compare the adherence level to MD (low, medium or high) both in the boys and in the girls concerning the distinct variables of body composition and physical fitness, one-way ANOVA (with Bonferroni post hoc) was done. The statistical significance was set as *p* <0.05. Cohen’s d was calculated to assess the Effect Size of differences. All the statistical analyses were done with the IBM SPSS statistics (version 23.0).

## 3. Results

Table 1 notes the values of the variables age, body composition, physical condition and KIDMED index of all the sample and differentiated by gender. In the analysis of the anthropometric variables we checked how, though the boys present statistically higher differences in the variables weight (+8.35%, *p* < 0.001, d = 0.326) and height (+2.48%, *p* < 0.001, d = 0.710), there were not statistically significant differences in the body mass index (BMI, p = 0.141). Moreover, the boys presented a lower body fat percentage (–9.06, *p* < 0.001, d = –1.078) but a significantly higher waist perimeter (+6.7%, *p* < 0.001, d = 0.471). As to the variables which make up the physical fitness, we checked that the boys had a significantly higher performance in each of the tests of the battery (*p* < 0.001). The adherence level for the whole sample (KIDMED index) is classified as medium (5.83 ± 2.32). Furthermore, we observed how there were no differences in the KIDMED index when making a comparison between genders (see Table 1).

Statistically significant differences were found between the boys and the girls in the alpha fitness index (*p* < 0.001) (see Table 1). When analyzing the proportion of the boys and the girls in each category of the Alpha Fitness battery test, 57.9% of the population was classified as Low level, followed by average with 28%, Very low (12.3%) and High (1.7%). Regarding the boys, 60.9% are in the low level, 24.7% in average, 13.1% very low and 1.3% in high. 86.3% of the girls are concentrated in low (54.9%) and average (31.4%) and the remaining 13.7% is divided into very low (11.5%) and high (2.2%).

When performing the descriptive analysis of adherence to the MD according to the age of the subjects, we observed that the adherence level decreases as the age of the participants in this research increases (*p* < 0.001), although in all cases they are at an average level of adherence to the MD according to the KIDMED index. The univariate GLM analysis did not show any statistically significant differences between boys and girls concerning adherence (F = 1.769; P = 0.184). In addition, as shown in Table 2, when making pair comparisons for all ages for both sexes, there were no differences between the sexes either. On the other hand, the differences in adherence to the MD are interesting with respect to age, since there were differences between the youngest and the oldest participants when referring to the entire sample, and there were differences between age and adherence in girls, but they were not statistically significant (see Table 2).

**Figure 1:**
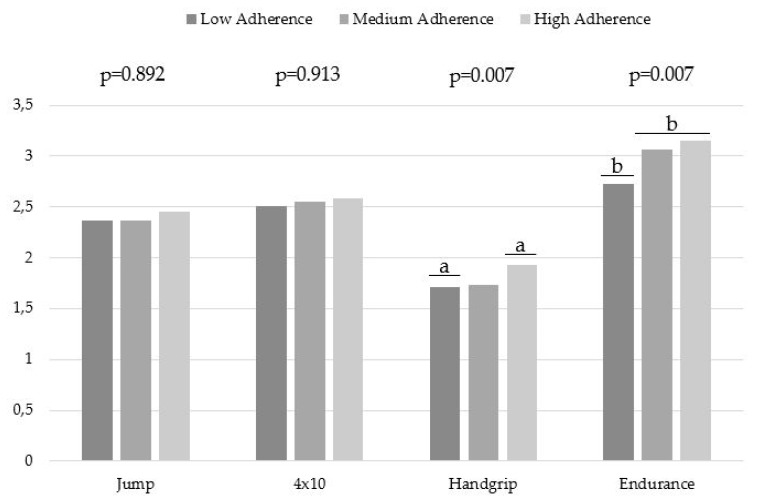
Alpha Fitness physical variables results based on the average levels established by Ortega et al. (2011) according to the different levels of adherence to the MD [39]. Note: Alpha Fitness Levels: 1 (very low), 2 (low), 3 (average), 4 (high), 5 (very high). ^a^ statistically significant difference between high adherence with respect to low, ^b^ statistically significant differences between medium and high adherence with respect to low.

Figure 1 shows the relationship between the different levels of adherence to the MD (low, medium and high) and the different levels established by Ortega et al. (2011) for tests of the physical condition of the Alpha Fitness battery (very low, low, average, high and very high) for the whole sample [39]. When comparing the classification of the average levels of the participants with a low adherence to the MD with the levels established by Ortega et al. (2011) [39], these were very low in handgrip, and low in jump, speed/agility and endurance; while those who followed a medium or high adherence were very low in handgrip, low in jumps and speed/agility (4 × 10), and medium in endurance. Although, there was a slight improvement in the data obtained in the physical fitness tests for the medium and high adherence to the MD group with respect to the low adherence group, there were no significant data except for the resistance test, in which adherence to the MD was shown to be a determinant (see Figure 1).

When carrying out a stratification based on the degree of adherence to the MD in the sample, the percentage of subjects who presented a low adherence was 16.79%, while 57.37% had an average level of adherence and 25.84% a high level of adherence. When comparing the values of the variables of body composition according to the degree of adherence to the MD, a statistically significant difference was detected in height in the girls. In the case of the boys, significant differences were not observed, except in their age. As to the tests which make up the physical fitness, it was found that both the boys and the girls showed a better performance in the test of endurance, when the subjects had a medium or high adherence with respect to low (*p* < 0.05). In the rest of the tests established to measure physical condition according to the Alpha Fitness battery, there was no difference according to the level of adherence to the MD by sex (see Table 3). 

## 4. Discussion

The current research is the first to analyze and describe the health-related physical fitness and adherence to the MD of Sevillian adolescents in the same research work. 

Once the participants’ body composition was analyzed, significant differences between genders were found. Differences in height, weight, fat percentage and waist circumference were obtained (see Table 1). These results are similar to those obtained in various studies done with a teenage population in which the women’s population presented a greater adiposity while the men’s population obtained higher values in weight, height and waist perimeter [36,40,41,42,43]. It is important to clarify that, in spite of significant differences existing in the aforementioned variables, participants of both sexes had medium BMI values, body fat percentage and waist perimeter. Furthermore, when relativizing the mean data of both genders at the different levels of body composition established by Moreno et al. (2006, 2007), the average BMI values, waist circumference (cm) and body fat percentage are classified as medium in each of the parameters [44,45].

Similarities were found when comparing the weight of all the population analyzed with the reference values contributed by Ortega et al. (2011) in which the weight, height and BMI in teenagers were transversally valued [39]. In contrast with what Wärnberg et al. (2006) obtained, there is no prevalence of obesity in the students, given that their values of body fat, BMI and waist circumference are considered normal [46]. The data found also vary from those obtained by Grosso et al. (2013), where nearly half of participants were overweight or obese with a mean BMI of 21.8 ± 3.1 [47]. 

Referring to the performance in the physical fitness tests, the results of this research present significant differences in the variables of manual dynamometry, long jump, 4 × 10m and endurance (see Table 1), as the boys got better results than the girls in each physical test (*p* < 0.01). These results are similar to those of other studies carried out [36,38,48,49,50]. This may be related to the results obtained in cross-sectional studies with European adolescents, where male adolescents were more active than girls [51], and this may result, somehow, in a better physical fitness. 

As mentioned previously, dietary patterns related to the MD have been considered as the healthiest, with numerous benefits, such as better mental and physical fitness status, among others [52,53,54,55]. The results found in the KIDMED questionnaire (poor 16.8%, medium 57.4% and high adherence 25.8%) are higher than those of similar studies carried out in Mediterranean countries [51,56,57]. Furthermore, and similar to the data reported by Grosso et al. (2013), no significant differences were found in adherence to the MD according to gender. Studies with a similar methodology but undertaken in non-Mediterranean countries present different results. Novak et al. (2017) reported worse final results in adolescents from Lithuania and Serbia, with a poor (39%), medium (47.7%) and high (13.3%) adherence to the MD, while Galan-Lopez (2018) found similar results (poor 14.99%, medium 60.72% and high 24.29% adherence to the MD) in Icelandic teenagers [36].

In general, the results obtained are similar to those of other studies conducted in southern European countries, in which the sample analyzed shows medium adherence to the MD [51,58,59]. Furthermore, the results of the KIDMED index agree with the results obtained in other studies of non-Mediterranean countries [36,55]. This research shows the need to reinforce the adherence to the MD in adolescents, since, as the subjects’ age increases, their adherence level decreases (see Table 2).

In contrast to the results of Ozen et al. (2015) [60], who obtained significant differences with respect to low and high adherence in the subjects analyzed, with a clear tendency to abandon the MD, our results show a clear tendency to maintain or increase the patterns related to this type of diet, since 83.2% of the participant population show medium or high adherence. In addition, as shown in Table 2, age was a determining factor in adherence to the MD in the total sample, being higher in younger ages. When only girls are considered, there were not any statistically significant differences when comparing different age groups (13- to 16-years-old) in pairwise comparisons. These results could be explained due to the combination of different factors, such as the stigmatization of obesity, the ideal of thinness and exaltation of the body, the evolution of gender stereotypes, and the aggressiveness of marketing mainly in women [61], although more studies would be needed to support this theory.

Recent publications directly relate the weight and the BMI with the adherence to the MD [55,62]. These data are different to those obtained, as, unlike the results found, the subjects present a similar adherence irrespective of a lesser or greater BMI and/or fat mass percentage. With respect to the relationship between waist circumference with adherence to the MD, Bacopoulou et al. (2017), conducted research with more than 1600 subjects of similar ages to those of the present study. They determined that an increase in adherence to the MD was associated with a decrease in waist perimeter, which indicates a potential school intervention to fight against abdominal obesity in adolescents. These data do not coincide with those found in the present study because, as occurs with the BMI and the fat mass percentage, the waist perimeter is not related with a lesser or greater adherence to the MD. When categorizing the medium levels with the classification established by Moreno et al. (2006, 2007), the subjects of both genders with distinct levels of adherence to the MD presented a medium level in the percentage variables of body fat and waist perimeter. On the contrary, when comparing the regulatory levels of the medium levels of these variables with the categorization established by Moreno et al. (2006, 2007), both genders present medium values in waist perimeter and the body fat percentage [39,40].

This research presents a novelty, as it compares the results of the tests related with physical fitness for health and the level of adherence to the MD of the participants, looking for possible associations (see Table 3). The same as the study carried out by Galán-López et al. (2018), it is confirmed that the participants with the highest performance in the physical condition tests (especially in cardiorespiratory endurance) are those with the greatest adherence to the MD (see Table 3). According to Lloyd and Oliver (2012), the greater development of cardiorespiratory fitness in relation with the rest of the components of physical fitness in children and adolescents maybe since it is the physical quality that is most worked in sport in school-age [63].

It is worth highlighting the significant differences observed in the performance of the endurance test in those boys and girls with low adherence to the MD with respect to those with a high adherence. These results are similar to those found by Galán-López et al. (2018), with the exception of the 4 × 10 m test, which has significant differences in the boys. These results concord with the conclusions of recent research works. The first of them positively related a high performance in the endurance test with a high adherence to the MD [55]. The second study not only demonstrated the relationship between high adherence to the MD and high levels of health-related physical fitness, but the subjects also showed optimal levels of health-related quality of life [52]. The importance of the level of physical fitness cannot be ignored and, therefore, nor can its evaluation, since it is directly linked to the health-related quality of life and inversely to early mortality. [64]. When categorizing the average levels of both genders at the distinct levels of physical condition established by Ortega et al. (2011) by the different levels of adherence to the MD [41], while the average value of the boys and the girls is established as a low value, the girls have a medium value except in endurance.

The disparity of the results according to gender can be explained by the different moments and levels of evolutive development. These can determine a greater performance by the boys in the physical fitness tests [65,66].

Observing Table 2, we also find notable differences by gender in all the physical fitness tests done, regardless of their adherence to the MD. However, as girls experience an earlier development than boys, this determines the capacity of developing higher levels of strength, speed and endurance [63], although these levels could also be due to girls being more sedentary and having higher physical inactivity with respect to boys. 

It is of great importance to address young people through educational interventions. This should be the basis that empowers people to make healthy decisions in their daily lives. The acquisition of knowledge and motivation through education, along with environmental support, will allow people to change unhealthy lifestyle habits [67].

This study’s cross-sectional design has limitations, as the contributions must not be attributed to plausible causes. These could be used as indications for coming research works. In addition, the data collection using the KIDMED questionnaire was self-reported, which could lead to an error in the reports and to memory bias due to the nature of the study.

## 5. Conclusions

The participants in this research showed a medium/low level of physical fitness level, with the girls obtaining slightly lower results than their male counterparts. Their adherence to the MD was classified as medium/high as 57% of them are at a medium level and 26% at a high level. The adherence levels (low, medium and high) showed a similar prevalence to those obtained by diverse studies undertaken in Spain and other Mediterranean countries with adolescent populations. Additionally, it appeared that age was a determining factor in adherence to the MD in the total sample, being lower as the age of the sample increases. This only applied to the male subjects when discriminating by sex. On the other hand, body composition was not affected by the level of adherence to the MD. As for the physical fitness test and compliance with the MD, there were significant differences in strength test results for those with high adherence versus those with low adherence and in resistance for those with a medium and high adherence to the MD versus those with a low adherence. 

As determinants of a good lifestyle, establishing the combination of a medium or high MD adherence with good physical fitness will result in a better health-related quality of life in adolescents. At the same time, more prospective cohort and intervention studies can elucidate better the relations between adherence to the MD and the results of health and behavior (physical condition). We call for the development of public health programs, campaigns of awareness and the creation of environments of practicing physical activity and healthy diets, as together they lead to an improvement of health better than when separated, hence the current importance of interdisciplinary interventions.

## Figures and Tables

**Table 1:** Anthropometric characteristics, physical fitness variables (Alpha Fitness Battery) and Mediterranean Diet Adherence (KIDMED). Inter-gender comparisons (M ± SD).

	Variables	Total (*n* = 917)	Boys (*n* = 458)	Girls (*n* = 459)	Inter-Gender *		Effect Size **
					F test	p value	d Cohen
Anthropometric	Weight (kg)	56.75 ± 13.48	59.00 ± 14.69	54.49 ± 11.73	27.52	<0.001	0.326
	Height (m)	1.67 ± 0.10	1.65 ± 0.11	1.61 ± 0.49	155.4	<0.001	0.710
	BMI (kg/m^2^)	21.67 ± 4.53	21.45 ± 4.16	21.89 ± 4.86	1.063	0.303	−0.069
	% Fat	22.36 ± 8.49	17.82 ± 7.76	26.88 ± 6.55	375.2	<0.001	−1.078
	Waist (cm)	72.01 ± 10.16	74.34 ± 10.84	69.68 ± 8.84	54,63	<0.001	0.471
Fitness	Handgrip (kg)	24.34 ± 6.50	27.07 ± 7.23	21.62 ± 4.19	234.5	<0.001	0.836
	Jump (cm)	149.24 ± 33.18	166.28 ± 31.96	132.02 ± 24.56	340.2	<0.001	1.026
	4 × 10 m (s)	12.55 ± 1.50	11.92 ± 1.39	13.19 ± 1.34	219.1	<0.001	−0.849
	Endurance (CRF)	5.15 ± 2.16	6.09 ± 2.37	4.22 ± 1.40	215.2	<0.001	0.867
	Alpha Index	2.64 ± 0.56	2.58 ± 0.58	2.70 ± 0.60	8.531	0.004	0.203
	KIDMED Index	5.83 ± 2.32	5.93 ± 2.31	5.73 ± 2.33	1.721	0.190	0.098
	Age (years)	14.83 ± 1.10	14.83 ± 1.11	14.83 ± 1.10		0.883	−0.009

M ± SD: Mean ± Standard Deviation; BMI: Body Mass Index; Waist: Waist Circumference; CRF: Cardiorespiratory Fitness; * p value: GLM (Age as covariate) with Bonferroni adjust. Student t-test to compare Age inter-gender. ** d Cohen: Effect Size (0.2 = Small, 0.5 Medium and 0.8 Large).

**Table 2:** Adherence to the MD (KIDMED Index, 0–12 points) by gender and age, (M ± SD).

	Years Old					Inter-Age Comparisons *		PartialEta^2^
	Total (*n*)	13 (*n*)	14 (*n*)	15 (*n*)	16 (*n*)	F test	p value	
Total	5.83 ± 2.32 (917)	6.32 ± 2.35 ^ab^ (222)	5.98 ± 2.36 ^c^ (262)	5.62 ± 2.24 ^a^ (238)	5.33 ± 2.22 ^bc^ (195)	7.27	<0.001	0.023
Boys	5.93 ± 2.32 (458)	6.54 ± 2.29 ^a^ (113)	5.97 ± 2.37 (131)	5.77 ± 2.2 (114)	5.37 ± 2.27 ^a^ (100)	4.81	0.002	0.016
Girls	5.73 ± 2.33 (459)	6.08 ± 2.39 (109)	5.98 ± 2.37 (131)	5.48 ± 2.28 (124)	5.29 ± 2.19 (95)	3.03	0.029	0.010

(M ± SD) = Means ± Standard Deviation; PartialEta^2^ = Partial Eta Squared * GLM Univariate Analysis of Variance (ANOVA), two factors (Age and Gender). Post hoc Bonferroni adjustment. The same superscripts (a,b,c) in values indicate significant differences inter age groups to pairwise comparisons. No significant differences to inter-gender comparisons were showed.

**Table 3:** Anthropometric characteristics and physical fitness variables of stratified according to adherence to the MD in both genders.

	Adherence to the Mediterranean Diet (KIDMED Index)							
	Boys				Girls			
	Low (*n* = 70/458)	Med (*n* = 262/458)	High (*n* = 126/458)	*p* Value	Low (*n* = 84/459)	Med (*n* = 264/459)	High (*n* = 111/459)	*p* Value
Age (years)	15.00 ± 1.04 ^a^	14.91 ± 1.15	14.57 ± 1.04	0.049 *	15.04 ± 1.09	14.85 ± 1.09	14.66 ± 1.11	0.062
Weight (kg)	61.78 ± 18.24	58.84 ± 14.19	57.90 ± 13.22	0.197	54.10 ± 12.42	54.91 ± 11.52	54.73 ± 9.65	0.851
Height (m)	1.67 ± 0.10	1.65 ± 0.11	1.65 ± 0.10	0.212	1.57 ± 0.62 ^a^	1.58 ± 0.07	1.60 ± 0.06	0.020 *
BMI (kg/m^2^)	21.87 ± 5.35	21.49 ± 4.14	21.14 ± 3.52	0.497	21.75 ± 4.44	21.87 ± 4.28	21.40 ± 3.59	0.596
% Fat	17.40 ± 8.72	18.25 ± 7.82	17.17 ± 7.06	0.385	26.69 ± 6.49	27.21 ± 6.63	26.48 ± 5.94	0.565
Waist (cm)	75.20 ± 12.34	74.61 ± 10.33	73.40 ± 9.49	0.437	69.60 ± 9.21	69.63 ± 9.19	69.86 ± 7.71	0.967
Handgrip (kg)	28.13 ± 7.37	26.77 ± 6.89	27.10 ± 7.81	0.377	21.37 ± 4.11	21.59 ± 3.93	22.29 ± 3.81	0.199
Jump (cm)	168.37 ± 28.94	164.91 ± 33.46	167.98 ± 30.40	0.568	131.11 ± 25.40	133.05 ± 23.77	131.14 ± 25.85	0.709
4 × 10 m (s)	11.79 ± 1.31	11.92 ± 1.36	11.99 ± 1.48	0.624	13.38 ± 1.40	13.12 ± 1.24	13.24 ± 1.50	0.259
Endurance (CRF)	5.59 ± 2.06 ^a^	6.07 ± 2.34	6.42 ± 2.56	0.048 *	3.82 ± 1.43 ^a^	4.29 ± 1.36	4.35 ± 1.42	0.014 *

Note: M ± SD = Medium ± Standard Deviation, BMI = Body Mass Index, Waist = Waist Circumference, CRF = Cardiorespiratory Fitness. * Express statistically significant differences between groups (*p* < 0.05). ^a^ Express statistically significant differences between Low with respect to Med and High.

## References

[1] Loef M., Walach H. (2012). The combined effects of healthy lifestyle behaviors on all-cause mortality: A systematic review and meta-analysis. Prev. Med..

[2] Tomba E. (2011). Assessment of lifestyle in relation to health. The Psychosomatic Assessment: Strategies to Improve Clinical Practice.

[3] Suárez-Carmona W., Sánchez-Oliver A.J., González-Jurado J.A. (2017). Pathophysiology of obesity: Current view. Rev. Chil. Nutr..

[4] Longmuir P., Colley R., Wherley V., Tremblay M. (2014). Risks and benefits of childhood physical activity. Lancet Diabetes Endocrinol..

[5] Tremblay M.S., Gray C.E., Akinroye K., Harrington D.M., Katzmarzyk P.T., Lambert E.V., Liukkonen J., Maddison R., Ocansey R.T., Onywera V.O. (2014). Physical Activity of Children: A Global Matrix of Grades Comparing 15 Countries. J. Phys. Act. Health.

[6] Tremblay M.S., Barnes J.D., González S.A., Katzmarzyk P.T., Onywera V.O., Reilly J.J., Tomkinson G.R., Team G.M. (2016). Global Matrix 2.0: Report Card Grades on the Physical Activity of Children and Youth Comparing 38 Countries. J. Phys. Act. Health.

[7] Ortega F.B., Ruiz J.R., Castillo M.J., Sjöström M. (2008). Physical fitness in childhood and adolescence: A powerful marker of health. Int. J. Obes..

[8] Ekelund U., Anderssen S.A., Froberg K., Sardinha L., Andersen L.B., Brage S., Group E.Y.H.S. (2007). Independent associations of physical activity and cardiorespiratory fitness with metabolic risk factors in children: The European youth heart study. Diabetologia.

[9] Janz K.F., Burns T.L., Levy S.M. (2005). Tracking of activity and sedentary behaviors in childhood: The Iowa bone development study. Am. J. Prev. Med..

[10] Serra-Majem L., Roman-Vinas B., Sanchez-Villegas A., Guasch-Ferre M., Corella D., La Vecchia C. (2019). Benefits of the Mediterranean diet: Epidemiological and molecular aspects. Mol. Asp. Med..

[11] Lake A.A., Mathers J.C., Rugg-Gunn A.J., Adamson A.J. (2006). Longitudinal change in food habits between adolescence (11–12 years) and adulthood (32–33 years): The ASH30 study. J. Public Health.

[12] Serra-Majem L., Roman B., Estruch R. (2006). Scientific evidence of interventions using the Mediterranean diet: A systematic review. Nutr. Rev..

[13] Martinez-Gonzalez M.A., Bes-Rastrollo M., Serra-Majem L., Lairon D., Estruch R., Trichopoulou A. (2009). Mediterranean food pattern and the primary prevention of chronic disease: Recent developments. Nutr. Rev..

[14] Schwingshackl L., Schwedhelm C., Galbete C., Hoffmann G. (2017). Adherence to mediterranean diet and risk of cancer: An updated systematic review and meta-analysis. Nutrients.

[15] Sofi F., Abbate R., Gensini G.F., Casini A. (2010). Accruing evidence about benefits of adherence to the Mediterranean diet on health: An updated systematic review and meta-analysis. Am. J. Clin. Nutr..

[16] Sofi F., Cesari F., Abbate R., Gensini G.F., Casini A., Transmission P.P. (2014). Adherence to Mediterranean diet and health status: Meta-analysis. BMJ.

[17] García Cabrera S., Herrera Fernández N., Rodríguez Hernández C., Nissensohn M., Román-Viñas B., Serra-Majem L. (2015). Prevalence of Low Adherence to the Mediterranean Diet in Children and Young; a Systematic Review. Nutr. Hosp..

[18] Iaccarino Idelson P., Scalfi L., Valerio G. (2017). Adherence to the Mediterranean Diet in children and adolescents: A systematic review. Nutr. Metab. Cardiovasc. Dis..

[19] Sanchez-Oliver A.J., Martín-García C., Gálvez-Ruiz P., González-Jurado J.A. (2018). Mortality and Economic Expenses of Cardiovascular Diseases Caused by Physical Inactivity in Spain. J. Phys. Educ. Sport.

[20] Sallis J.F., Glanz K. (2009). Physical activity and food environments: Solutions to the obesity epidemic. Milbank Q..

[21] Palomino-Devia C., Reyes-Oyola F.A., Sánchez-Oliver A. (2018). Levels of physical activity, health-related quality of life, physical self-concept and body-mass index among Colombian students. Biomedica.

[22] Twig G., Yaniv G., Levine H., Leiba A., Goldberger N., Derazne E., Ben-Ami Shor D., Tzur D., Afek A., Shamiss A. (2016). Body-Mass Index in 2.3 Million Adolescents and Cardiovascular Death in Adulthood. N. Engl. J. Med..

[23] (2015). Organización Mundial de la Salud Estrategia Mundial para la Salud de la Mujer, el Niño y el Adolescente (2016–2030).

[24] Khatib O. (2004). Noncommunicable diseases: Risk factors and regional strategies for prevention and care. East. Mediterr. Health J..

[25] Lobstein T., Baur L., Uauy R. (2004). Obesity in children and young people: A crisis in public health. Obes. Rev. Suppl..

[26] Popkin B.M. (2006). Global nutrition dynamics: The world is shifting rapidly toward a diet linked with noncommunicable diseases. Am. J. Clin. Nutr..

[27] Waters E., de Silva-Sanigorski A., Burford B.J., Brown T., Campbell K.J., Gao Y., Armstrong R., Prosser L., Summerbell C.D. (2011). Interventions for preventing obesity in children. Cochrane Database Syst. Rev..

[28] Wolfenden L., Jones J., Williams C.M., Finch M., Wyse R.J., Kingsland M., Tzelepis F., Wiggers J., Williams A.J., Seward K. (2016). Strategies to improve the implementation of healthy eating, physical activity and obesity prevention policies, practices or programmes within childcare services. Cochrane Database Syst. Rev..

[29] Suárez-Carmona W., Sánchez-Oliver A.J. (2018). Índice de masa corporal: Ventajas y desventajas de su uso en la obesidad. Relación con la fuerza y la actividad física. Nutr. Clín. Med..

[30] (2017). NCD Risk Factor Collaboration (NCD-RisC) Worldwide trends in body-mass index, underweight, overweight, and obesity from 1975 to 2016: A pooled analysis of 2416 population-based measurement studies in 128.9 million children, adolescents, and adults. Lancet.

[31] Sawyer S.M., Afifi R.A., Bearinger L.H., Blakemore S.-J., Dick B., Ezeh A.C., Patton G.C. (2012). Adolescence: A foundation for future health. Lancet.

[32] Serra-Majem L., Ribas L., Ngo J., Ortega R.M., García A., Pérez-Rodrigo C., Aranceta J. (2004). Food, youth and the Mediterranean diet in Spain. Development of KIDMED, Mediterranean Diet Quality Index in children and adolescents. Public Health Nutr..

[33] Serra-Majem L., García-Closas R., Ribas L., Pérez-Rodrigo C., Aranceta J. (2001). Food patterns of Spanish schoolchildren and adolescents: The enKid Study. Public Health Nutr..

[34] Cabrera S.G., Fernández N.H., Hernández C.R., Nissensohn M., Román-Viña B., Serra-Majem L. (2015). Test KIDMED; prevalencia de la Baja Adhesión a la Dieta Mediterránea en Niños y Adolescentes; Revisión Sistemática. Nutr. Hosp..

[35] Ruiz J.R., Castro-Piñero J., España-Romero V., Artero E.G., Ortega F.B., Cuenca M.A.M., Enez-Pavón D.J., Chillón P., Girela-Rejón M.J., Mora J. (2011). Field-based fitness assessment in young people: The ALPHA health-related fitness test battery for children and adolescents. Br. J. Sports Med..

[36] Galan-Lopez P., Ries F., Gisladottir T., Domínguez R., Sánchez-Oliver A.J. (2018). Healthy Lifestyle: Relationship between Mediterranean Diet, Body Composition and Physical Fitness in 13 to 16-Years Old Icelandic Students. Int. J. Environ. Res. Public Health.

[37] Léger L.A., Mercier D., Gadoury C., Lambert J. (1988). The multistage 20 metre shuttle run test for aerobic fitness. J. Sports Sci..

[38] Villa-González E., Ruiz J.R., Chillón P. (2015). Associations between active commuting to school and health-related physical fitness in spanish school-aged children: A cross-sectional study. Int. J. Environ. Res. Public Health.

[39] Moreno L.A., Mesana M.I., González-Gross M., Gil C.M., Ortega F.B., Fleta J., Wärnberg J., León J.F., Marcos A., Bueno M. (2007). Body fat distribution reference standards in Spanish adolescents: The AVENA Study. Int. J. Obes..

[40] Ortega F., Artero E., Ruiz J., España-Romero V., Jiménez-Pavón D., Vicente-Rodriguez G., Moreno L., Manios Y., Béghin L., Ottevaere C. (2011). Physical fitness levels among European adolescents: The HELENA study. Br. J. Sports Med..

[41] Barker A.R., Gracia-Marco L., Ruiz J.R., Castillo M.J., Aparicio-Ugarriza R., González-Gross M., Kafatos A., Androutsos O., Polito A., Molnar D. (2018). Physical activity, sedentary time, TV viewing, physical fitness and cardiovascular disease risk in adolescents: The HELENA study. Int. J. Cardiol..

[42] Joensuu L., Syväoja H., Kallio J., Kulmala J., Kujala U.M., Tammelin T.H. (2018). Objectively measured physical activity, body composition and physical fitness: Cross-sectional associations in 9- to 15-year-old children. Eur. J. Sport Sci..

[43] Gualteros J.A., Torres J.A., Umbarila-Espinosa L.M., Rodríguez-Valero F.J., Ramírez-Vélez R. (2015). A lower cardiorespiratory fitness is associated to an unhealthy status among children and adolescents from Bogotá, Colombia. Endocrinol. Nutr..

[44] España-Romero V., Artero E.G., Santaliestra-Pasias A.M., Gutierrez A., Castillo M.J., Ruiz J.R. (2008). Hand Span Influences Optimal Grip Span in Boys and Girls Aged 6 to 12 Years. J. Hand Surg. Am..

[45] Moreno L.A., Mesana M.I., González-Gross M., Gil C.M., Fleta J., Wärnberg J., Ruiz J.R., Sarría A., Marcos A., Bueno M. (2006). Anthropometric body fat composition reference values in Spanish adolescents. The AVENA Study. Eur. J. Clin. Nutr..

[46] Syväoja H.J., Kankaanpää A., Kallio J., Hakonen H., Kulmala J., Hillman C.H., Pesonen A.-K., Tammelin T.H. (2018). The Relation of Physical Activity, Sedentary Behaviors, and Academic Achievement Is Mediated by Fitness and Bedtime. J. Phys. Act. Health.

[47] Wärnberg J., Ruiz J.R., Ortega F.B., Romeo J., González-Gross M., Moreno L.A., García-Fuentes M., Gómez S., Nova E., Díaz L.E. (2006). Estudio AVENA (Alimentación y valoración del estado nutricional en adolescentes). Resultados obtenidos 2003–2006. Pediatría Integr..

[48] García-Sánchez A., Burgueño-Menjibar R., López-Blanco D., Ortega F.B. (2013). Condición física, adiposidad y autoconcepto en adolescentes. Estudio piloto. Rev. Psicol. Deport.

[49] Secchi J.D., García G.C., España-Romero V., Castro-Piñero J. (2014). Condición física y riesgo cardiovascular futuro en niños y adolescentes argentinos: Una introducción de la batería ALPHA. Arch. Argent. Pediatr..

[50] Fernández I., Canet O., Giné-Garriga M. (2017). Assessment of physical activity levels, fitness and perceived barriers to physical activity practice in adolescents: Cross-sectional study. Eur. J. Pediatr..

[51] Grosso G., Marventano S., Buscemi S., Scuderi A., Matalone M., Platania A., Giorgianni G., Rametta S., Nolfo S., Galvano S. (2013). Factors associated with adherence to a Mediterranean diet among adolescents living in Sicily, southern Italy. Nutrients.

[52] Evaristo O.S., Moreira C., Lopes L., Abreu S., Agostinis-Sobrinho C., Oliveira-Santos J., Póvoas S., Oliveira A., Santos R., Mota J. (2018). Associations between physical fitness and adherence to the Mediterranean diet with health-related quality of life in adolescents: Results from the LabMed Physical Activity Study. Eur. J. Public Health..

[53] Bacopoulou F., Landis G., Rentoumis A., Tsitsika A., Efthymiou V. (2017). Mediterranean diet decreases adolescent waist circumference. Eur. J. Clin. Investig..

[54] Novak D., Štefan L., Prosoli R., Emeljanovas A., Mieziene B., Milanović I., Radisavljević-Janić S. (2017). Mediterranean Diet and Its Correlates among Adolescents in Non-Mediterranean European Countries: A Population-Based Study. Nutrients.

[55] Muros J.J., Cofre-Bolados C., Arriscado D., Zurita F., Knox E. (2017). Mediterranean diet adherence is associated with lifestyle, physical fitness, and mental wellness among 10-y-olds in Chile. Nutrition.

[56] Martino F., Puddu P.E., Pannarale G., Colantoni C., Zanoni C., Martino E., Barillà F. (2014). Metabolic syndrome among children and adolescents from Southern Italy: Contribution from the Calabrian Sierras Community Study (CSCS). Int. J. Cardiol..

[57] Peng W., Goldsmith R., Berry E.M. (2017). Demographic and lifestyle factors associated with adherence to the Mediterranean diet in relation to overweight/obesity among Israeli adolescents: Findings from the Mabat Israeli national youth health and nutrition survey. Public Health Nutr..

[58] Santomauro F., Lorini C., Tanini T., Indiani L., Lastrucci V., Comodo N., Bonaccorsi G. (2014). Adherence to Mediterranean diet in a sample of Tuscan adolescents. Nutrition.

[59] Grosso G., Galvano F. (2016). Mediterranean diet adherence in children and adolescents in southern European countries. NFS J..

[60] Ozen A.E., Bibiloni M.D.M., Murcia M.A., Pons A., Tur J.A. (2015). Adherence to the Mediterranean diet and consumption of functional foods among the Balearic Islands’ adolescent population. Public Health Nutr..

[61] Toro J., Gila A., Castro J., Pombo C., Guete O. (2005). Body image, risk factors for eating disorders and sociocultural influences in Spanish adolescents. Eat Weight Disord..

[62] Mistretta A., Marventano S., Antoci M., Cagnetti A., Giogianni G., Nolfo F., Rametta S., Pecora G., Marranzano M. (2017). Mediterranean diet adherence and body composition among Southern Italian adolescents. Obes. Res. Clin. Pract..

[63] Lloyd R.S., Oliver J.L. (2012). The youth physical development model: A new approach to long-term athletic development. Strength Cond. J..

[64] De Moraes A.C.F., Vilanova-Campelo R.C., Torres-Leal F.L., Carvalho H.B. (2019). Is Self-Reported Physical Fitness Useful for Estimating Fitness Levels in Children and Adolescents? A Reliability and Validity Study. Medicina.

[65] Damian M., Oltean A., Damian C. (2018). The Impact of sedentary behavior on health and the need for physical activity in children and adolescents. Rev. Rom. Pentru Educ. Multidimens..

[66] Schutte N.M., Nederend I., Hudziak J.J., de Geus E.J.C., Bartels M. (2016). Differences in Adolescent Physical Fitness: A Multivariate Approach and Meta-analysis. Behav. Genet..

[67] Đorđić V., Božić P., Milanović I., Radisavljević S., Batez M., Jorga J., Ostojić S.M. (2019). Guidelines-Driven Educational Intervention Promotes Healthy Lifestyle Among Adolescents and Adults: A Serbian National Longitudinal Study. Medicina.

